# The Philosophy of Storms

**Published:** 1842-02

**Authors:** 


### REVIEWS.

---

Art. III.—*The Philosophy of Storms.* By James P. Espy, A. M., Member of the American Philosophical Society, and Corresponding Member of the National Institution, Washington. Boston: Charles C. Little & James Brown. 1841. pp. 552.

Meteorology is a subject towards which no one can feel an entire indifference. So much and so constantly is our comfort affected by the weather, that all persons are concerned in understanding the laws which regulate its changes. In the work before us, Mr. Espy treats at great length, and, in our judgment, with great ability, of some of the meteors which have awakened among men the most general and absorbing interest. His theory of storms has been before the public for a number of years, and has attracted the attention of scientific men as well in Europe as in our own country. By some it has been favorably received, and by others it has been rejected; so that much high authority might be cited both for and against the doctrine. We have said that the subject appears to us to have been handled with great ability, by which we do not wish to be understood as expressing our full assent to the truth of the theory, but as saying that the author has fortified it by a powerful array of facts and arguments. We are free to go farther and declare, that no other theory extant, appears to us to account so satisfactorily for the phenomena of storms. Mr. Espy's work consists of tracts prepared at different periods, and designed for separate publication, each containing a synopsis of the views of the author; in consequence of which much repetition occurs, and the volume has been swelled to a size which will deter many readers from such an examination as is necessary to doing the theory justice. We propose, therefore, for the satisfaction of such of our readers as may lack the opportunity or leisure for consulting the work, to present in this article an outline of Mr. Espy's philosophy of storms, in maturing which, he informs us, he has been engaged now about thirteen years—his attention having been called to the subject by Dr. Dalton's experiments on the aqueous vapor which exists in the atmosphere. "I was struck," he says, "with one of his results, namely, that the quantity of vapor in weight, existing at any time in a given space, could be determined with great accuracy in a few minutes, by means of a thermometer and a tumbler of water cold enough to condense on its outside a portion of the vapor in the air. It occurred to me at once, that this was the lever by which the meteorologist was to move the world."

This aqueous vapor, which is only five-eights of the specific gravity of atmospheric air, forms a restless and variable constituent of it, as it has been recently deposited in rain, or has been accumulating long in time of drought. When the air near the surface of the earth becomes more heated, or more highly charged with aqueous vapor, its equilibrium is disturbed and up-moving columns or streams will be formed. As they ascend, the upper parts of these columns coming under less pressure, will expand, the air of which they consist spreading out and assuming a mushroom shape. The air thus expanding, according to a well known law of caloric, will grow colder, at the rate of about one degree and a quarter for every hundred yards of its ascent. This decline in its temperature will necessarily be attended with the condensation of its aqueous vapor, or the formation of cloud. Mr. Espy illustrates this by an instrument which he calls a Nephelescope, in which a cloud of condensed vapor is formed in a glass bottle, the air of which, having been compressed by a syringe, is suddenly relieved of its pressure, and expands to its natural volume. The vapor contained in the air is condensed, as we see it, by the first strokes of the air-pump in a glass receiver, by the cold of diminished pressure; and the mercurial column appended to the nephelescope, shows that the condensation of this vapor is attended with an evolution of heat.

"The distance or height to which the air will have to ascend before it will become cold enough to begin to form cloud, is a variable quantity, depending on the number of degrees which the dew-point is below the temperature of the air; and this height may be known at any time by observing how many degrees a thin metallic tumbler of water must be cooled down below the temperature of the air before the vapor begins to condense on the outside. The highest temperature at which it will condense, which is variable, accordingly as there is more or less vapor in the air, is called the "dew-point," and the difference between the dew-point and the temperature of the air in degrees, is called the complement of the dew-point."

A warm air coming up from the south, after a severe frost in winter has its moisture condensed into fog by the cold earth. If it be cooled 20° below the dew-point, it will lose about one half of its vapor by condensation, and at 40° below, it will condense three-fourths of its vapor into water. The cold produced from the expansion of the up-moving columns will not, however, be followed by exactly these results; for the vapor itself growing thinner under the diminished pressure, the dew-point falls about one-quarter of a degree for every hundred yards of ascent.

"It follows, then, as the temperature of the air sinks about one degree and a quarter for every hundred yards of ascent, and the dew-point sinks about a quarter of a degree, that as soon as the column rises as many hundred yards as the complement of the dew-point contains degrees of Fahrenheit, cloud will begin to form; or, in other words, the bases of all clouds forming by the cold of diminished pressure from up-moving columns of air, will be about as many hundred yards high as the dew-point is below the temperature of the air at the time. If the temperature of the ascending column should be 10° above that of the air through which it passes, and should rise to the height of 4,800 feet before it begins to form cloud, the whole column would then be one hundred feet of air lighter than surrounding columns; and if the column should be very narrow, its velocity of upward motion would follow the laws of spouting fluids, which would be eight times the square root of one hundred feet a second; that is, eighty feet a second, and the barometer in the centre of the column at its base would fall about the ninth of an inch."

The rate of cooling is slower after the cloud begins to form, and proceeds only half as rapidly above the base of the cloud as below it. The air growing colder one degree for every hundred yards of ascent, the air in the cloud above its base loses but five-eights of a degree for every hundred yards in height; and hence, if the cloud be of great perpendicular height, its summit must be much warmer, and consequently much lighter, than the surrounding atmosphere. Gay Lussac found this to be the fact—his thermometer rising when he passed out of the clear sunshine into a cloud. The heating effect of condensation is still greater when the change is from vapor to hail or snow. With the evolution of heat by this change, the ascending column is expanded, taking the shape already adverted to; and immediately under the cloud the barometer, consequently, will fall; whilst it will rise above the mean under the annulus formed by the outspreading of the air in the top of the cloud. The air which thus raises the mercury in the barometer must, from its greater weight, sink downwards and increase the velocity of the wind at the surface of the earth, towards the centre of the ascending column. The up-moving current of air, is consequently, supplied by the air within the annulus, and that which descends within the annulus itself.

"When up-moving currents are formed by superior heat, clouds will more frequently begin to form in the morning, increase in number as the heat increases, and cease altogether in the evening, when the surface of the earth becomes cold by radiation."

The wind increases with the increase of these columns, both keeping pace with the increasing temperature.

The circumstances under which rain is not likely to occur, are detailed in the following paragraph:

"When the complement of the dew-point is very great, (29° and more,) clouds can scarcely form; for up-moving columns will generally either come to an equilibrium with the surrounding air, or be dispersed before they rise twenty hundred yards, which they must do, in this case, before they form clouds. Sometimes, however, masses of air will rise high enough to form clouds; but they are generally detached from any moving column underneath, and cannot form cumuli with flat bases; such clouds will be seen to dissolve as soon as they form, and even while forming they will generally appear ragged, thin, and irregular. Moreover, if the ground should be colder during the day than the air in contact with it, as it sometimes happens after a dry cold spell of weather, then, as the air touching the cold earth will be colder than the stratum above it, ascending columns cannot exist, and of course no cumuli can be formed on that day, even though the air may be saturated with vapor to such a degree as to condense a portion of it on cold bodies at the surface of the earth. Also, if during the whole winter, any part of Siberia or the northern part of North America, should be so much colder than surrounding regions, that no up-moving columns could be produced, then neither clouds nor snow could be formed. Neither can clouds form of any very great size when there are cross currents of air sufficiently strong to break in two an ascending current; for the ascensional power of the up-moving current will thus be weakened and destroyed. Immediately after a great rain, too, when the upper air has yet in it a large quantity of caloric, which it received from the condensation of the vapor, the up-moving columns which may then occur, on reaching this upper stratum, will not continue their motion in it far, from the want of buoyancy; therefore, they will not produce rain, nor clouds of any kind but broken cumuli. Besides, the air at some distance above the surface of the earth, and below the base of the cloud, is sometimes very dry, and as much of this air goes in below the base of the cloud and up with the ascending column, large portions of the air in the cloud may thus not be saturated with vapor, and, of course, rain in this case will not be produced. These are some of the means contrived by nature to prevent up-moving columns from increasing until rain would follow. Without some such contrivances, it is probable that every up-moving column which should begin to form cloud when the dew-point is favorable, would produce rain; for as soon as cloud forms, the up-moving power is rapidly increased by the evolution of the caloric of elasticity."

On the leeward side of very lofty mountains it is known that rain never falls, and we have an explanation of the fact in the extract just presented. The air on the windward side as it rises up the brow of the mountains, loses its vapor by condensation; or even if enough moisture remain to form clouds, these, as they begin to descend the leeward slope, coming under greater pressure, are dissolved by the heat thus produced.

We come now to our author's philosophy of the tornado and water-spout.

"If the air is very hot below, with a high dew-point, and no cross currents of air above to a great height, then, when an up-moving current is once formed, it will go on and increase in violence as it acquires perpendicular elevation, especially after the cloud begins to form. At first, the base of the cloud will be flat; but after the cloud becomes of great perpendicular diameter, and the barometer begins to fall considerably, as it will do, from the specific levity of the air in the cloud, then the air will not have to rise so far as it did at the moment when the cloud began to form, before it reaches high enough to form cloud from the cold of diminished pressure. The cloud will now be convex below, and its parts will be seen spreading outwards in all directions, especially on that side towards which the upper current is moving, assuming something of the shape of a mushroom. In the meantime, the action of the in-moving current below, and up-moving current in the middle, will become very violent, and if the barometer falls two inches, under the centre of the cloud, the air, on coming in under the cloud, will cool by diminished pressure about 10°, and the base of the cloud will reach the earth, if the dew-point was only 8° below the temperature of the air at the time the cloud began to form. The shape of the cloud will now be that of an inverted cone, with its apex on the ground, and when a little more prolonged and fully developed, it will be what is called a tornado, if it is on land, and a water-spout if at sea."

In proof of these positions the author refers to the appearances presented along the path of a tornado, where the trees and other objects will show that the wind blew from all points towards the centre of the path. The trees on the extreme borders will all be found prostrated with their tops inwards, either inwards and backwards, or inwards and forwards, or exactly transverse to the path of the tornado. The trees in the centre of the path will be thrown either backwards or forwards, or parallel to the track; "and invariably if one tree lies across another, the one which is thrown backwards is underneath." Materials will be carried across the path, those which were on the right hand making a curve from left to right, and those on the left hand from right to left. The limbs of trees near the borders of the path are found twisted round the trees, and broken in such a manner as to remain twisted, those on the right side of the path from left to right, and those on the opposite side from right to left. The limbs only that grew on the side of the tree most distant from the track of the tornado, are broken, as they alone are subjected to the cross-strain.

While the inmoving currents are driving all objects towards the path of the tornado, in the centre of the path bodies are exposed to a different action. The air at that point, suddenly relieved of its accustomed pressure, expands with instant and immense force. Houses will be either wholly exploded, by the expanding air, their walls being prostrated outwards, or else have their roofs blown up, and those most nearly air-tight will be most subject to demolition. Doors and windows will be blown out, floors from cellars will be blown up, and bureaus and corks of empty bottles exploded. The thin sealed bottle under the exhausted receiver, shivered into fragments by the expansive force of the confined air, beautifully illustrates this principle, and the history of the tornado at Natchez, which we refer to as one of the most recent, is full of evidences of the truth of it. Mr. Espy relates a multitude of such instances, and has, to our mind, fully established this part of his theory.

"All round the tornado, at a short distance, probably not more than three or four hundred yards, there will be a dead calm, on account of the annulus formed by the rapid efflux of the air above, from the centre of the upmoving and expanding column."

In this calm atmosphere the barometer stands above the mean, showing a depression of the atmosphere at that point. Beyond the annulus a gentle wind blows from the tornado, and hence all the air which feeds it must be supplied from within the annulus. Light bodies, such as shingles, branches of trees, and drops of rain, must be carried to a great height by these ascending columns; "for though they may frequently be thrown outwards above, and then descend to a considerable distance at the side they will meet with an inblowing current below, which will force them back to the upmoving current, and so they will be carried aloft again." In this manner hail will be formed, and we have no where met with so satisfactory an explanation of this meteor. The rain carried into the region of perpetual frost will be congealed, especially if it be thrown out above so far as to fall into clear air, which, in some instances, is from 30° to 40° colder than the air in the cloud. The hail from a column of air ascending perpendicularly, will be thrown out on both sides of the tornado's path, sloping inwards as it falls, and on examination it will be found that two veins of hail fall simultaneously, at no great distance apart. This hail will frequently be found to contain particles of matter, carried up from the surface of the earth with the ascending column of air; and in cases of violent action sheets of water will be raised above the snow line, where they will form cakes of ice, and descending, will break into every variety of shape.

"In the Orkney spout of the 24th July, 1818, instead of hail-stones of the usual shape, pieces of ice, of almost all forms, were precipitated with the utmost violence. Mr. Caithness attempted to wade out among the hail-stones in the direction of the cattle, but the loose ice, he says, slipped below his feet, and sometimes reached his knees. In this way his legs were so much cut by its sharp edges, that he was soon obliged to desist.

The barometer fell from 29.68 to 27.76, on the passage of the spout; or perhaps more, as the minimum may not have been observed; for it did not occur to Mr. Lindsay to note the barometer till the cloud was passing off; sixty geese in one flock were killed, and all the rest so hurt that they soon died; and the milch cows "were struck yeld," or gave no more milk, and indeed would not suffer the people to attempt to milk them any more."

In all violent thunder-storms in which hail falls, our author maintains that the drops of rain are carried by the ascending air into the region of eternal ice, and we think with him, that "it is difficult, if not impossible, to conceive any other way in which hail can be formed in the summer, or in the torrid zone."

The author copies a chart from the memoirs of the French Academy, representing two veins of hail which fell simultaneously not more than eight minutes at any one place, travelling from the Pyrenees to the Baltic, with a velocity of about 50 miles an hour. Between the veins of hail rain fell throughout the whole extent of the storm.

"In those countries in which an upper current of air prevails in a particular direction, the tornadoes and water spouts will generally move in the same direction, because the upmoving column of air in this meteor rises far into this upper current, and of course its upper part will be pressed in this direction, and as the great tornado cloud moves on in the direction of the upper current, the air at the surface of the earth will be pressed up into it by the superior weight of the surrounding air. It is for this reason that the tornado in Pennsylvania generally moves toward the eastward."

As air cannot move upwards without coming under diminished pressure, and as it must thus grow cooler by its expansion, and thus give rise to cloud, any cause which produces an upward vortex of air, whether it be a volcano or a great conflagration, will induce rain, provided the complement of the dew-point be small, the air calm below and above, and the upper part of the atmosphere of its ordinary temperature. Mr. Espy remarks,

"Let all these favorable circumstances be watched for in time of drought (and they can only occur then,) and let the experiment be tried."

The experiment is to kindle a conflagration, in which the warm air by ascending will grow cold, and condensing its vapor, induce rain. If the experiment should succeed, and we should thus be put in possession of a practicable mode of averting tornadoes and bringing on rain in times of drought, we agree with our author, that "the result would be highly beneficial to mankind." The experiment may not be successful, or if so, it may prove too expensive to be resorted to as an ordinary expedient; but this would not affect the truth of Mr. Espy's theory, which, although objections may be urged against it, has the merit of being simple and ingenious, and of accounting more satisfactorily than any other proposed, for the phenomena of storms. It does not take into account the action of electricity in these meteors, and in this respect the author himself admits that his theory is incomplete. When completed, as he proposes, by the study of the influence of electricity, we think, with the Committee of the French Academy of Science, "he will leave nothing to be desired," in this department of philosophy. Y.

